# Fertilization Changes Chemical Defense in Needles of Mature Norway Spruce (*Picea abies*)

**DOI:** 10.3389/fpls.2018.00770

**Published:** 2018-06-07

**Authors:** Line Nybakken, Marit H. Lie, Riitta Julkunen-Tiitto, Johan Asplund, Mikael Ohlson

**Affiliations:** ^1^Faculty of Environmental Sciences and Natural Resource Management, Norwegian University of Life Sciences, Ås, Akershus, Norway; ^2^Department of Environmental and Biological Sciences, University of Eastern Finland, Joensuu, Finland

**Keywords:** nitrogen, fertilization, phenolics, chemical defense, *Picea abies*, spruce, conifers

## Abstract

Nitrogen availability limits growth in most boreal forests. However, parts of the boreal zone receive significant levels of nitrogen deposition. At the same time, forests are fertilized to increase volume growth and carbon sequestration. No matter the source, increasing nitrogen in the boreal forest ecosystem will influence the resource situation for its primary producers, the plants, with possible implications for their defensive chemistry. In general, fertilization reduces phenolic compound concentrations in trees, but existing evidence mainly comes from studies on young plants. Given the role of the phenolic compounds in protection against herbivores and other forest pests, it is important to know if phenolics are reduced with fertilization also in mature trees. The evergreen Norway spruce is long-lived, and it is reasonable that defensive strategies could change from the juvenile to the reproductive and mature phases. In addition, as the needles are kept for several years, defense could also change with needle age. We sampled current and previous year needles from an N fertilization experiment in a Norway spruce forest landscape in south-central Norway to which N had been added annually for 13 years. We analyzed total nitrogen (N) and carbon (C), as well as low-molecular phenolics and condensed tannins. Needles from fertilized trees had higher N than those from controls plots, and fertilization decreased concentrations of many flavonoids, as well as condensed tannins in current year needles. In previous year needles, some stilbenes and condensed tannins were higher in fertilized trees. In control trees, the total phenolic concentration was almost five times as high in previous year needles compared with those from the current year, and there were great compositional differences. Previous year needles contained highest concentrations of acetophenone and stilbenes, while in the current year needles the flavonoids, and especially coumaroyl-astragalins dominated. Condensed tannins did not differ between current and previous year needles from control trees. In conclusion, the phenolic defense of current year needles of mature *P.abies* trees was strongly changed upon fertilization. This may imply that nitrogen deposition and forest fertilization leave forests less robust in a time when pests may take advantages of a changing climate.

## Introduction

Boreal and temperate forests soils naturally have a low availability of nitrogen (N) and N is often the primary growth-limiting nutrient in these soils (Tamm, 1991; Binkley and Fisher, 2013). However, the increased nitrogen deposition rates over the last 150 years have greatly altered this in parts of these biomes (Galloway and Cowling, 2002; Meunier et al., 2016), and it is well-known that N deposition often directly enhances forest growth (Meunier et al., 2016). Continental and northern regions of the European boreal forests, however, are relatively little affected by N deposition (e.g., Gundale et al., 2011). Yet, there is increasing interest in fertilization of these areas (e.g., Bergh et al., 2014) to increase timber volumes, and more recently, as a measure to sequester more carbon (C) (see e.g., Gundale et al., 2014).

No matter the source for the increased N availability, the consequences for the ecosystem are potentially large. N deposition has been shown to increase C uptake by trees in the range of 15–60 kg of C per kg of N deposited (Högberg et al., 2006; Pregitzer et al., 2008; Thomas et al., 2010; de Vries et al., 2014; Gundale et al., 2014). N deposition may also increase tree foliar N concentration by 22% for conifers and 13% for deciduous trees (reviewed by Throop and Lerdau, 2004). The general power of N is pointed out in the recent review by Meunier et al. (2016), who conclude that increased availability of N in the base of forest food webs results in cascade effects, which in turn substantially affect other trophical levels through alterations in food web structure and functioning.

Forest fertilization was originally introduced to increase the vitality of forests, and indeed, most studies show an increase in diameter growth after fertilization (e.g., Bergh et al., 2014). At present, however, many plant pathogens are increasing in incidence and range (Parmesan, 2006; Tylianakis et al., 2008), and it is possible that elevated N-availability is one of the drivers behind this increase. Simultaneously, N deposition has been shown to have a positive effect on insect herbivore populations (e.g., Strengbom et al., 2005), increasing the individual performance and population growth rates by up to 30% (Throop and Lerdau, 2004). Fertilization increased the palatability of bilberry (*Vaccinium myrtillus*) to gray sided voles (Strengbom et al., 2003).

As N availability increase the tree's C requirements for growth, it may also influence the C availability for other metabolic processes with indirect implications for ecosystem functioning. For instance, trees produce large amounts of C-based plant secondary metabolites (PSMs). One PSM group of major ecological interest is the phenolics, with functions as diverse as herbivory defense (e.g., Bryant et al., 1983; Coley et al., 1985), pathogen resistance (Witzell and Martín, 2008), allelopathy or symbioses signaling (Inderjit, 1996; Mandal et al., 2010), frost and drought hardiness (Samanta et al., 2011), and photodamage protection (Lois, 1994; Close and McArthur, 2002). Flavonoids, stilbenes, phenolic acids, and condensed tannins are important classes of phenolics in most forest trees. According to ecological theory, the levels of C-based PSMs in plants could be expected to change with increased N. The carbon nutrient balance (CNB) hypothesis (Bryant et al., 1983), and the related growth differentiation hypothesis (GDB) Loomis, 1932; Herms and Mattson, 1992, in short postulate that the restrictions on growth caused by low N availability will lead to an accumulation of photosynthates, which can be diverted to the production of PSMs. The protein competition model (PCM) (Jones and Hartley, 1999) further claims that the underlying mechanism is that production of proteins and phenolic compounds compete for their common precursor phenylalanine. These hypotheses are corroborated in a meta-analysis by Koricheva et al. (1998), who found that plant phenolics in general decrease after fertilization. Consistent with these hypotheses, another meta-analysis showed that species adapted to resource-rich habitats grow inherently faster and invest less in defenses than species adapted to less productive habitats (Endara and Coley, 2011).

Nevertheless, surprisingly few studies have tried to test experimentally how increased N availability affects the defensive capacity of species from inherently N limited ecosystems. Of these, only a handful involve the ecologically and economically important boreal conifers. Virjamo et al. (2014) observed an increase in catechins in the needles after fertilization of 1-year old *Picea abies* seedlings. All other phenolics stayed unchanged. Blodgett et al. (2005) and Edenius et al. (2012) found reduced concentrations of total needle phenolics in fertilized *Pinus resinosa* and *P.abies*, respectively. On the other hand, Kytö et al. (1996) saw no effect of fertilization on the total phloem phenolics of *P.abies*. These previous studies have either focused on very young trees (Virjamo et al., 2014), a mixture of different aged trees and needles (Edenius et al., 2012), or have only looked at the effect on total phenolics (Kytö et al., 1996; Blodgett et al., 2005), while individual compounds or groups of compounds have not been studied. This implies that there is very little scientific knowledge on how increased N availability may influence the within-plant variation in defensive capacity. Fertilization may possibly increase growth, but leave the tree more subject to attacks from different forest herbivores or other pests. Most boreal conifers keep their needles for two or more years, and use their needles as storage compartments for carbohydrates and nutrients, with generally highest concentrations in young needles (see e.g., Ohlson, 1995 and references therein). The total levels increase and the composition of PSMs change with needle age over one season (Slimestad and Hostettmann, 1996; Slimestad, 1998; Ganthaler et al., 2017a), as well as upon infection by a rust fungus (Ganthaler et al., 2017b). However, we lack information about how such dynamics in defensive strategies e.g., over needle age, are affected by abiotic environmental changes.

Here we report on phenolic concentrations, as well as total C and N from current and previous year needles of mature spruce (*P.abies*), fertilized over a 13-year period. We hypothesized that increased N availability would increase N concentrations in the needles, and lead to decreased levels of PSMs, but also affect individual compounds differently, resulting in compositional changes. We further hypothesized that previous year needles would have higher defense levels and be less subject to change upon fertilization than those from the current year.

## Material and methods

### Study area and fertilization experiment

Our study area was an old and multilayered boreal Norway spruce forest landscape situated 800 m a s l near Kittilbu in SE Norway (61° 10′N, 09° 09′E). The spruce trees varied in size and age from seedlings to 20 m tall and 220 year old trees. No logging have occurred during the last 65 years, but light selective loggings have occurred in the past. The ground vegetation was dominated by bilberry *V. myrtillus* and bryophytes such as e.g., *Pleurozium schreberi, Hylocomium splendens*, and *Polytrichum commune*. Gauslaa et al. (2008) and Davey et al. (2017) gives information about the vegetation and the edaphic and climatic conditions in the study area.

Nitrogen has been added annually since 2003 to ten 15 × 15 m (225 m^2^) experimental plots in the forest landscape at a rate of 150 kg ha^−1^ year^−1^ in the form of granulated pellets that contained 24.6% N, 2% P, 6% K, and trace elements (YaraMila Fullgjødsel). Ten equal sized and unfertilized plots were established to serve as control. Individual plots were located between 50 and 350 m from the next plot. Background deposition of N for the years 2007–2011 amounts to ~6 kg ha^−1^ year^−1^ (http://www.environment.no/topics/air-pollution/acid-rain/maps-deposition-of-sulphur-and-nitrogen/).

### Chemical extraction

We sampled spruce needles on 25 June 2016 in paper bags containing silica. From each plot, we chose three approximately equal sized and visually vital trees, from the dominating crown layer. From these c. 10 needles from both the current and the previous year's cohorts were sampled. The needle samples were taken ~1.2 m above the ground and from the north side of the tree, to get as little variation as possible due to light exposition. The paper bags were brought to the lab the same day, and were dried in an oven at 30°C for 48 h and thereafter stored in plastic bags in the freezer at −18°C. The plant material was later grinded to fine powder using a Retsch MM400 ball mill (Retsch, Haag, Germany) at 30 revolutions s^−1^ for 30–180 s. From the resulting powder, we determined total carbon (C) and nitrogen (N) with a Micro Cube (Elementar Analysen, Hanau, Germany), using 5–6 mg plant material. For phenolic analysis, further sub-samples of *c*. 10 mg were extracted with 600 μl methanol (MeOH) and homogenized with 3–4 zirconium oxide balls at 5,000 rpm for 20 s on a Precellys 24 homogeniser (Bertin Technologies, Montigny-le-Bretonneux, France). Samples were then cooled on ice for 15 min before being centrifuged at 15,000 rpm min^−1^ for 3 min (Eppendorf centrifuge 5417C, Eppendorf, Hamburg, Germany). The supernatant was transferred to a 10 ml glass tube with a Pasteur pipette. The residue was again dissolved in 600 μl MeOH, homogenized, and centrifuged in the same manner as above; the supernatant was removed, and the same extraction process was conducted two more times until both the residue and the supernatant was completely colorless. The combined supernatants were evaporated in a vacuum centrifuge (Eppendorf concentrator plus; Eppendorf, Hamburg, Germany), sealed, and stored in a freezer (−18°C) until high performance liquid chromatography (HPLC) analysis. The residues were also stored in a freezer for further analysis on MeOH—insoluble condensed tannins.

### HPLC analyses

The dried extracts were dissolved in 200 μl MeOH with the help of an ultra sonic cleaner (mod. no. USC200TH; VWR International LLC, Randor, USA) and diluted with 200 μl ultra-clean water (USF ELGA Maxima HPLC; Veolia Water Technologies, Saint-Maurice, France). Samples were poured into 2 ml Eppendorf tubes and centrifuged at 15000 rounds min^−1^ for 3 min before being forced through a syringe filter (GHP Acrodisc 13 mm Syringe Filter with a 0.45 μm GHP membrane; PALL Corporation, Washington, USA) and sealed inside HPLC vials. Low molecular weight phenolics (LMWP) were analyzed using a HPLC system (Agilent Series 1200, Agilent Technologies, Waldbronn, Germany) with a G1312A binary pump, a G1329A autosampler, a G1316A thermoregulated column heater, and a G1315D diode array detector. As the stationary phase a Thermo Scientific column type was used (Thermo Fisher Scientific Inc., Waltham, USA), with a 50 × 4.6 mm internal diameter and filled with ODS Hypersil (3 μm) particles. The mobile phase consisted of two solvents that eluted the samples by way of a gradient as in Julkunen-Tiitto and Sorsa (2001). The injection volume was 20 μl. The phenolic compounds were identified using a UHPLC quadrupole time-of-flight liquid chromatograph/mass spectrometer (UHPLC/Q-TOF MS) (6540 series, Agilent) (Supplementary Table S1; Virjamo et al., 2014). The eluents were 1.5% tetrahydrofuran + 0.25% acetic acid in Milli-Q ultrapure water (Eluent A) and 100% MeOH (Eluent B). The following gradient was used for eluent A: 0–1.5 min 100% A, 1.5–3 min 100–85% A, 3–6 min 85–70% A, 6–12 min 70–50% A, 12–20 min 50% A, 20–22 min 50–0% A. The flow rate was 0.4 ml min^−1^ and the injection volume was 0.5 μl. The Q-TOF parameters were: mass range 100–3,000 m/z; temperature of the drying gas and sheath gas 350°C and flow rates 12 l/min and 11 l/min, respectively; nebulizer pressure 35 psi; capillary voltage 3,500 V; nozzle voltage 1,000 V; fragmentor voltage 80 V; skimmer voltage 65 V; octopole voltage 750 V. The reference m/z 922.0098 was used for accurate mass measurements. Accuracy of tentative compound identification (ppm) was calculated as (measured mass—calculated mass) × 10^6^/calculated mass (Supplementary Table S1). Compounds that were not identifiable using the Q-TOF MS were identified by comparing retention times and ultraviolet spectra to the literature.

The absorption spectra at 270 and 320 nm, along with respective retention times, were used to identify the chemical compounds and to calculate concentrations by comparing with commercial standards.

### Analysis of condensed tannins (CT)

Concentrations of both MeOH-soluble and MeOH-insoluble CTs were identified using the acid butanol assay for proanthocyanidins described in Hagerman (2002). The HPLC-vials were removed from the auto sampler maximum 48 h after analysis and from these 50–100 μl were used to determine the amounts of MeOH-soluble CTs. The amount of MeOH-insoluble CTs were analyzed from the residues left after the extraction process. The samples were put in 10 ml glass tubes along with enough MeOH to equal 0.5 ml in total (0.5 ml MeOH regardless for MeOH-insoluble tannins), then further mixed with 3 ml butyric acid (95% butanol, 5% hydrochloric acid), and 100 μl iron reagent (2 M HCL with 2% ferric ammonium sulfate). The glass tubes were properly sealed, mixed, and placed in boiling water for 50 min. Duplicate samples was prepared when extract amounts allowed. After cooling, the light absorption at 550 nm was determined using a spectrophotometer (UV-1800; Shimadzu Corp., Kyoto, Japan). The average between duplicate samples was used as one data value. Purified tannins from spruce needles were used as standards to calculate concentrations.

### Data analysis

For each compound, we used Split plot ANOVAs to test for the effect of treatment as main plot factor and age as sub-plot factor. The composition of phenolic compounds in the needles were visualized with the two first axes of a principle component analysis (PCA) using the R package vegan (Oksanen et al., 2016). We used the ordiellipse function (Oksanen et al., 2016) to plot the 95% confidence intervals (CI; based on SE-values) of the respective age × treatment centroid. All analyses were performed using R 3.2.5 (R Core Team, 2018).

## Results

### Total N and C concentrations

**Figure 1:**
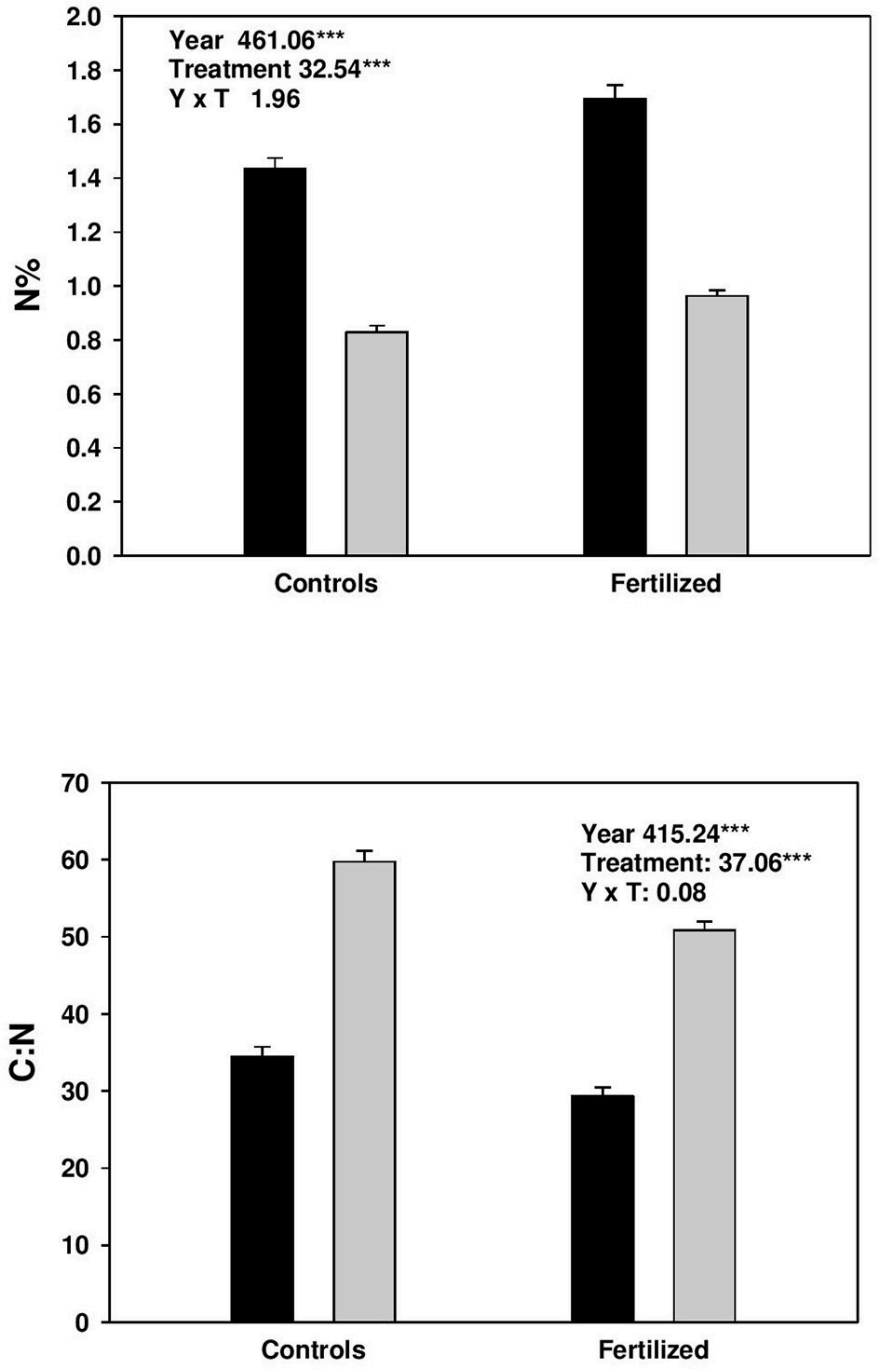
Concentration (%) of nitrogen (N) and C:N in current (black bars) and previous year needles (gray bars) from controls and fertilized plots. Split plot ANOVAs on nitrogen concentration and C:N of needles. The effect of treatment is the main plot factor and year (age) is the sub-plot factor. The data was sqrt-transformed.

Fertilization increased N% in both current and previous year needles, and correspondingly decreased the C:N ratio (Figure 1). At the same time, N% was higher, and C: N ratio lower in current year needles compared with those from the previous year (Figure 1).

### Phenolic concentrations

**Figure 2:**
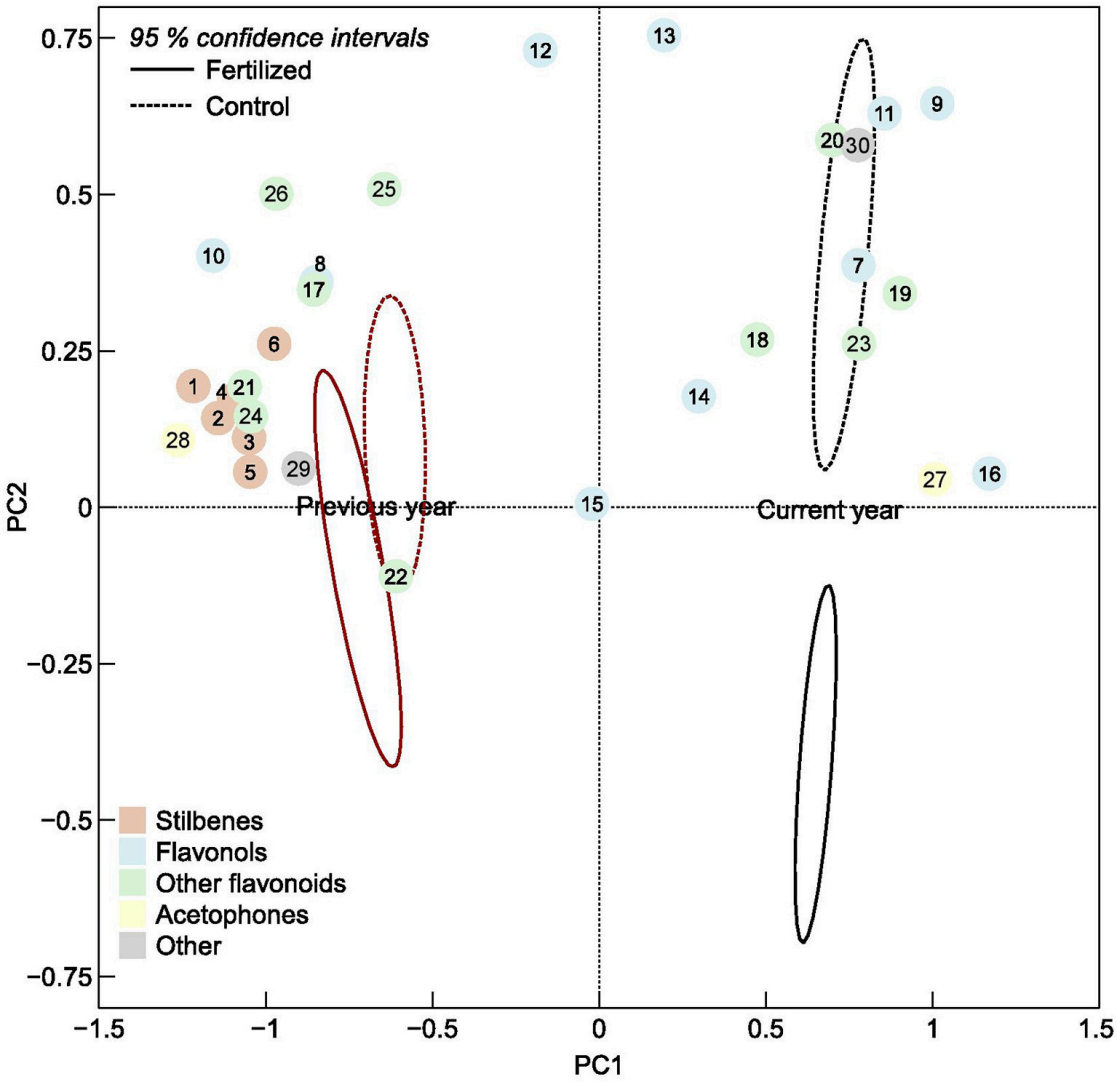
Ordination plot of the first and second principal component scores of individual phenolic compounds, numbered according to Table 1, in current and previous year's spruce needles. The ellipses are 95% confidence intervals of the fertilized plots (solid line) and control plots (dotted lines), respectively.

Control needles from the previous year contained over three times more total low molecular phenolics than did current year needles (Table 1), and the composition differed greatly (Figure 2, Table 1). In previous year needles (controls), stilbenes constituted 33% of the total low molecular phenolics concentration, and the most abundant compounds were piceatannol glucoside and resveratrol glucoside. In comparison, stilbenes represented only 0.03% in current year needles. On the other hand, current year needles had more than double concentration of flavonols compared with the older needles, of which 3,6-dicoumaroyl astragalin was the highest compound in both needle types (Table 1). The other flavonols were present in minor concentrations, mostly differing significantly between the two needle cohorts, but in both directions. The same was true for most other flavonoids, but (+)-catechin and gallocatechin had more than 2 and 10 times higher concentration in previous year needles, respectively. We identified picein only in current year needles, while the older needles had more than 30 times more its aglycone, 4-hydroxy acetophenone (Table 1). In addition, the needle cohorts contained two different unknown lignans and the same three procyanidins as well as B_3_ in minor concentrations. Control needles from the previous year contained about the same amount of the two fractions of condensed tannins, while the current year ones had higher concentration of the insoluble fraction.

**Table 1:** Concentrations (mg g^−1^ DW) (mean values ±1 SE) of and Split plot ANOVAs on phenolic compounds.

	**Current year needles**		**Previous year needles**		**Age (A)**	**Treatment (T)**	**A × T**
	**Controls**	**Fertilized**	**Controls**	**Fertilized**	***F*_1, 56_ (*P*)**	***F*_1, 18_ (*P*)**	***F*_1, 56_ (*P*)**
**STILBENES**							
(1) Piceatannol glucoside^†^	0.52 ± 0.06	0.36 ± 0.11	14.95 ± 0.98	13.88 ± 1.35	**1082.14 (<0.001)**	**13.44 (0.002)**	**9.55 (0.003)**
(2) Piceatannol aglycon^†^	0.15 ± 0.02	0.12 ± 0.03	4.76 ± 0.46	5.60 ± 0.64	**1296.00 (<0.001)**	0.67 (0.424)	**6.78 (0.012)**
(3) Resveratrol glucoside^†^	0.28 ± 0.03	0.12 ± 0.02	8.78 ± 1.00	13.44 ± 1.67	**639.60 (<0.001)**	2.53 (0.129)	**26.87 (<0.001)**
(4) Resveratrol aglycon	–	–	1.00 ± 0.12	1.85 ± 0.20	–	**10.25 (0.005)**	–
(5) Methylpiceatannol glucoside	–	–	1.05 ± 0.13	1.55 ± 0.22	–	**5.24 (0.034)**	–
(6) Methylpiceatannol aglycon^†^	–	–	0.77 ± 0.12	0.95 ± 0.17	–	0.33 (0.571)	–
*Sum, stilbenes*^‡^	*0.95 ± 0.10*	*0.60 ± 0.14*	*31.32 ± 1.87*	*37.48 ± 2.63*	***1148.56 (<0.001)***	*0.68 (0.421)*	***6.71 (0.012)***
**FLAVONOIDS**							
(7) Quercetin 3-galactoside^‡^	0.18 ± 0.03	0.06 ± 0.02	–	–	–	**5.48 (0.031)**	–
(8) Quercetin 3-glucuronide^‡^	0.09 ± 0.01	0.05 ± 0.01	0.62 ± 0.11	0.84 ± 0.16	**155.27 (<0.001)**	0.37 (0.550)	3.25 (0.077)
(9) Quercetin 3-glucoside^‡^	0.77 ± 0.06	0.55 ± 0.04	0.28 ± 0.03	0.15 ± 0.02	**153.25 (<0.001)**	**17.24 (<0.001)**	0.52 (0.473)
(10) Myricetin 3-galactoside	0.06 ± 0.03	0.02 ± 0.01	0.53 ± 0.03	0.58 ± 0.06	**241.11 (<0.001)**	0.00 (0.980)	1.46 (0.232)
(11) Kaempferol 3-glucoside^†^	0.99 ± 0.13	0.68 ± 0.1	0.26 ± 0.02	0.19 ± 0.01	**157.29 (<0.001)**	**8.13 (0.011)**	0.28 (0.601)
(12) Kaempferol 3-rhamnoside^†^	0.16 ± 0.06	0.10 ± 0.05	0.58 ± 0.04	0.43 ± 0.04	**21.88 (<0.001)**	2.40 (0.082)	0.03 (0.855)
(13) Isorhamnetin glucoside	0.61 ± 0.05	0.53 ± 0.03	0.58 ± 0.04	0.43 ± 0.04	**4.35 (0.042)**	**4.85 (0.041)**	1.04 (0.313)
(14) Monocoumaroylastragalin	0.50 ± 0.07	0.58 ± 0.07	0.48 ± 0.05	0.35 ± 0.04	**7.28 (0.001)**	0.33 (0.572)	**7.91 (0.007)**
(15) 3,6dicoumaroylastragalin der	0.12 ± 0.01	0.13 ± 0.00	0.14 ± 0.01	0.12 ± 0.01	0.66 (0.420)	0.22 (0.647)	**4.58 (0.037)**
(16) 3,6 dicoumaroylastragalin	11.47 ± 0.73	11.08 ± 0.48	3.70 ± 0.11	3.22 ± 0.14	**338.72 (<0.001**)	0.61 (0.444)	0.01 (0.916)
*Sum, flavonols*	*15.01 ± 0.73*	*13.88 ± 0.58*	*6.83 ± 0.22*	*6.04 ± 0.30*	***344.31 (<0.001)***	*1.57 (0.227)*	*0.09 (0.771)*
(17) Luteolin aglycon	0.06 ± 0.03	–	0.22 ± 0.03	0.22 ± 0.04	–	0.01 (0.929)	–
(18) Unknown flavonoid^‡^	0.43 ± 0.05	0.57 ± 0.08	0.26 ± 0.04	0.33 ± 0.03	**12.26 (<0.001)**	3.91 (0.064)	0.02 (0.880)
(19) Apigenin 7-glucoside^‡^	0.38 ± 0.01	0.21 ± 0.06	–	–	–	**4.47 (0.048)**	–
(20) Apigenin aglycon^‡^	0.11 ± 0.01	0.06 ± 0.01	0.03 ± 0.01	0.03 ± 0.01	**40.42 (<0.001)**	**4.91 (0.040)**	3.25 (0.077)
(21) (+)-catechin	2.16 ± 0.15	0.97 ± 0.14	4.79 ± 0.23	4.30 ± 0.25	**282.71 (<0.001)**	**11.63 (0.003)**	3.89 (0.054)
(22) Gallocatechin^†^	1.96 ± 0.43	1.88 ± 0.27	10.84 ± 2.16	9.78 ± 2.14	**86.55 (<0.001)**	0.01 (0.916)	0.54 (0.464)
(23) B_3_^‡^	2.74 ± 0.53	2.48 ± 0.54	–	–	–	0.01 (0.928)	–
(24) Procyanidin 1	0.57 ± 0.04	0.41 ± 0.06	1.71 ± 0.16	1.67 ± 0.16	**173.61 (<0.001)**	**4.63 (0.045)**	0.00 (0.954)
(25) Procyanidin 2^†^	0.46 ± 0.06	0.16 ± 0.06	0.23 ± 0.02	0.23 ± 0.02	**116.34 (<0.001)**	**5.79 (0.027)**	**34.33 (<0.001)**
(26) Procyanidin 3^‡^	0.59 ± 0.13	0.44 ± 0.05	1.06 ± 0.10	1.03 ± 0.12	**28.79 (<0.001)**	0.087 (0.772)	0.015 (0.904)
*Sum, flavonoids*^†^	*21.75 ± 0.90*	*18.58 ± 0.75*	*26.88 ± 2.22*	*24.38 ± 2.24*	*2.07 (0.159)*	*2.94 (0.104)*	*0.09 (0.763)*
**ACETOPHENONES**							
(27) Picein^†^	0.31 ± 0.04	0.29 ± 0.03	–	–	–	0.00 (0.958)	–
(28) 4-hydroxy acetophenone	0.95 ± 0.12	0.78 ± 0.19	32.03 ± 1.16	29.37 ± 1.76	**833.23 (<0.001)**	1.65 (0.215)	1.44 (0.235)
*Sum, acetophenones*	*1.26 ± 0.13*	*1.03 ± 0.18*	*32.03 ± 1.16*	*29.37 ± 1.76*	***810.94 (<0.001)***	*1.69 (0.210)*	*1.40 (0.242)*
**OTHERS**							
(29) Lignan 1	–	–	3.38 ± 0.62	2.92 ± 0.53	–	1.57 (0.226)	–
(30) Lignan 2^†^	0.81 ± 0.13	0.20 ± 0.07	–	–	–	25.45 (<0.001)	–
*Sum, low molecular phenolics*	*27.50 ± 1.24*	*22.93 ± 1.16*	*93.61 ± 3.13*	*94.14 ± 4.03*	***595.87 (<0.001)***	*0.69 (0.416)*	*0.50 (0.483)*
**CONDENSED TANNINS**							
MeOH soluble	10.89 ± 1.77	4.08 ± 1.55	14.63 ± 1.45	25.49 ± 1.57	**45.50 (<0.001)**	0.78 (0.400)	**23.00 (<0.001)**
MeOH insoluble	41.87 ± 2.85	39.76 ± 3.11	33.51 ± 1.44	43.48 ± 1.53	1.65 (0.205)	2.51 (0.131)	**12.27 (<0.001)**
*Sum, condensed tannins*	*52.76 ± 3.26*	*48.68 ± 3.75*	*48.03 ± 2.23*	*68.63 ± 2.14*	**5.68 (0.022)**	**6.77 (0.018)**	**25.67 (<0.001)**

*The effect of treatment is the main plot factor and age is the sub-plot factor. Numbering of compounds is corresponding to numbering in the PCA (Figure 2)*.

† *log-transformed*,

‡ *sqrt-transformed. Numbers in bold indicate statistical significant results. Lines in italics indicate sums of chemical groups*.

The composition of phenolic compounds changed upon fertilization in both the current and previous year's needles (Figure 2), but this was statistically significant only for the new needles. However, the pattern differed between the two age classes. The total concentration of stilbenes increased upon fertilization in previous year needles, while it decreased in current year needles. These patterns were seen for all three individual stilbenes that were present in both needles cohorts (significant A × T interactions, Table 1). Two of three (resveratrol aglycon and methylpiceatannol glucoside) stilbenes that were only present in the older needles increased upon fertilization. Of the flavonols, quercetin 3-galactoside, quercetin 3-glucoside, kaempferol 3-glucoside, and isorhamnetin decreased upon fertilization in both needle cohorts. Monocoumaroylatragalin and the 3,6-dicoumaroylastragalin, on the other hand, increased in current year needles and decreased in those from the previous year (significant A × T interactions, Table 1). The flavonoids apigenin 7-glucoside, apigenin aglycon and (+)-catechin, as well as two of the procyanidins decreased in fertilized plots in both needle types.

The soluble fraction of condensed tannins changed strongly upon fertilization in both needle cohorts, decreasing in current year needles, while it increased in the older ones. The insoluble fraction, on the other hand, decreased slightly in the youngest needles, while it increased in those from the previous year (significant A × T interactions, Table 1).

## Discussion

Changes in the availability of a limiting resource such as nitrogen may have large impacts on ecosystem composition and functioning. In the present study, we showed that heavy fertilization increased the N concentrations of spruce needles, and induced changes in both concentrations and compositions of phenolic compounds.

The most striking result is the differences between the two needle cohorts. The current year needles reacted to fertilization in accordance with our first hypothesis and ecological theories on balance between C and N (e.g., Bryant et al., 1983; Jones and Hartley, 1999), as concentrations of many phenolic compounds were lower in needles from fertilized plots, compared with controls (Table 1). The difference was strongest for the soluble fraction of condensed tannins, which were reduced to less than half the concentration of controls. This decrease corresponds well with the decrease in (+)-catechin, the precursor of condensed tannins. In addition, many flavonoids had lowest concentrations in fertilized plots, as well as the total concentration of low molecular phenolics. The coumaroyl astragalins, however, was not affected by fertilization, suggesting high priority of these compounds in the young needles. Blodgett et al. (2005) and Edenius et al. (2012) also found reductions in total phenolics in fertilized *P. resinosa* and *P.abies*. Virjamo et al. (2014), on the other hand, found little effects of fertilization in needles of 1-year old *P.abies* seedlings, except that the concentration of (+)-catechin increased, also contrasting our results.

As expected, the previous year needles were less affected, but interestingly some individual stilbenes (and stilbenes in total), as well as both fractions of condensed tannins, were present in highest concentrations in needles from fertilized trees. It may be that increased N availability increased chlorophyll content and thus photosynthesis and C availability of fertilized trees. According to the carbon-nutrient balance hypothesis and the PCM (Bryant et al., 1983; Jones and Hartley, 1999, respectively), such an improved resource situation would be expected to give increased growth and not defense. Since defense was reduced in current year needles, this also challenges the established truth that new needles have priority when resources are distributed (resources are transported from old parts to young parts). Or, alternatively, it suggests that the defensive compounds of old needles cannot be transported, or dissolved/turned over and then transported, to younger needles.

As we hypothesized, previous year needles from control plots had higher concentrations of PSMs than the new needles. In addition, the composition of compounds differed strongly. The large picture was that the older needles accumulated large amounts of stilbenes and acetophenone, in addition to flavonoids, while the defensive system of new needles mostly consisted of flavonoids (Table 1). The levels of condensed tannins, however, did not differ much with age in needles from unfertilized trees. These results fits well with studies of seasonal changes in phenolics of *P.abies*. Both Slimestad (1998) and Ganthaler et al. (2017a) noted that flavonoids (and especially flavonols) were predominant in the first weeks after bud burst, after which stilbenes, picein and shikimic acid increased. Increases in stilbene concentrations toward late summer and autumn was also seen in some older studies (Kaufmann et al., 1974; Bjørnseth, 1977; Solhaug, 1990). Our results from previous year needles (sampled 1 year after they sprouted) suggest that the high protection levels achieved last autumn are kept over time. Flavonoids, and especially flavonols, are often found in the vacuoles of epidermal cells, where they play a role in light protection (Close and McArthur, 2002). Quercetins and kaempferols are often induced in response to enhanced levels of ultraviolet light (e.g., Ryan et al., 1998; Nybakken et al., 2012). In an experiment excluding ultraviolet (UVB) light from young scots pine plants (*Pinus sylvestris*), Turunen et al. (1999) showed that non-acetylated flavonol 3-glycosides were reduced, while the di-acetylated flavonols (dicoumaroyl astragalins) were unaffected. Earlier indoor studies, however, showed that also di-acetylated flavonols were induced by UVB radiation (Jungblut et al., 1995). It may be that light protection must be prioritized during early needle development in spring, when the epidermis is still thin and physical defenses like waxes are not so prominent. The high levels of dicoumaroyl astragalins, and their high resilience against change shown both here and by Turunen et al. (1999), suggest they are especially important in young conifer needles. Flavonoids may also play other defense roles, e.g., against herbivores (e.g., Bryant et al., 1983), and may be regarded as a general defense. Interestingly, the newly developed needles also contained high levels of the more complex flavonoids condensed tannins, which are also important herbivore defenses (Barbehenn and Constabel, 2011).

Stilbene levels, on the other hand, were very low in young needles. Both stilbenes and flavonoids are produced through the phenylpropanoid pathway from the precursor phenylalanine, but in the next step the enzymes stilbene synthase (STS) and chalcone synthase (CHS), respectively, lead to stilbene and flavonoid biosynthesis from the same cinnamoyl-CoA/p-coumaroyl-CoA precursor (Kodan et al., 2002). Based on this, we have no indications that stilbenes are more complex or costly to produce for the young needles, although this is scarcely studied. However, it may be that stilbenes serve a more specialized role, and are therefore less prioritized during early spring when resources (both C and N) are scarce. Stilbenes are located in the central part of spruce needles (Solhaug, 1990), as well as in the xylem of conifers (e.g. Harju et al., 2009), and should thus have other roles than light protection. Stilbenes are indeed expected to play roles in fungal protection, as they are frequently induced by pathogen attack (Chong et al., 2009; Jeandet et al., 2010; Ganthaler et al., 2017a), and varying concentrations have been associated with intraspecific variation of host plant susceptibility to infection (Lieutier et al., 2003). In addition to pathogens, the previous year needles most likely contained a higher amount of fungal endophytes, as they have been exposed to environmental inoculum for a longer time period. Little is known about how these microbes affect the biochemistry of plants, but they could potentially both consume, process or produce phenolics themselves (e.g., Hardoim et al., 2015; Yang et al., 2016).

To sum up, fertilization changed phenolic profiles of both current and previous year needles, but in different ways. Older needles partly increased their protection levels, both regarding what is thought to be specific fungal defense (stilbenes) as well as specific herbivore defense (condensed tannins). The new needles, on the other hand, showed reductions in the already low stilbene levels, as well as in condensed tannins and flavonoids. This may have implications for the susceptibility of spruce against attacks from fungal pathogens. Typically, needle diseases like rust attack the new needles, and a connection between phenolic levels and susceptibility to infection was found for spruce attacked by the needle bladder rust (*Chrysomyxa rhododendri*) (Ganthaler et al., 2017b). In sub-alpine spruce forests in central Europe, repeated infections by rust fungi has led to reduced timber yields and severe problems with regeneration of the species (Oberhuber et al., 1999; Ganthaler et al., 2014). Repeated rust (*C. abietis*) attacks on spruce are also observed in Scandinavia the latest years, but to our knowledge the impact of these are not yet studied. A changing climate with milder winters, as well as increased trade of living plants (Liebhold et al., 2012) may help pathogens to increase in abundance and outbreak frequencies, but also to reach new areas. The same is expected for insect pests. In a review, Throop and Lerdau (2004) concluded that N deposition has positive effects on general insect performance, probably though the effect it has on plants: increased N content and decreased PSMs. A study along latitudinal gradients in Europe showed that the load of sap-feeding insects on forest trees are driven by climatic patterns, indicating that also climate warming will drive plant losses to insect feeding (Kozlov et al., 2005). In conclusion, this may imply that nitrogen deposition and forest fertilization leave forests less robust in a time when pests may take advantages of a changing climate.

## Author contributions

LN planned and supervised the chemical analyses, interpreted the data and wrote the MS. ML designed the experiment, supervised the sampling, and contributed to writing the MS. JA conducted the statistical analyses and contributed to writing the MS. RJ-T supervised the UHPLC-MS analyses and the interpretation of data, and contributed to writing the MS. MO designed the experiment, performed the fertilizations, and contributed to writing the MS.

### Conflict of interest statement

The authors declare that the research was conducted in the absence of any commercial or financial relationships that could be construed as a potential conflict of interest.
