# Supplementary material for: Fertilization Changes Chemical Defense in Needles of Mature Norway Spruce (Picea abies)
Source: Front Plant Sci. 2018 Jun 7;9:770. doi: 10.3389/fpls.2018.00770 (PMC6000156; doi:10.3389/fpls.2018.00770)
Supplement: Supplementary file 1 [file Table_1.DOCX]

Supplementary Table S1. UHPLC-qtof identification of soluble non-tannin phenolic compounds

| LC Rt | Compound | M^+^ measured | M^+^ calculated | Accuracy (ppm) | Needles |
| --- | --- | --- | --- | --- | --- |
| 2.59 | lignan 1 | * |  |  | old |
| 2.77 | picein | * |  |  | new |
| 4.38 | gallocatechin | 305.0675 (M-H) | 305.0661 | 4.31 | old |
| 6.39 | B3 | 577.1366 (M-H) | 577.1346 | 3.47 | new |
| 8.87 | 4-hydroxy acetophenone | 135.0458 (M-H) | 135.0446 | 8.81 | both |
| 9.50 | (+)-Catechin | 291.0862 (M+H) | 291.0868 | -2.06 | both |
| 10.19 | procyanidin 1 | * |  |  | both |
| 11.46 | procyanidin 2 | * |  |  | both |
| 12.07 | procyanidin 3 | * |  |  | both |
| 13.10 | piceatannol glucoside (astringin) | 405.1210 (M-H) | 405.1186 | 6.02 | old |
| 13.47 | resveratrol glucoside | 405.1208 (M-H) | 405.3792 | -10.48 | old |
| 17.06 | piceatannol (aglycon) | 243.0603 (M-H) | 243.0658 | 22.63 | old |
| 17.88 | resveratrol (aglycon) | * |  |  | old |
| 17.50 | myricetin 3-galactoside | * |  |  | new |
| 20.12 | quercetin 3-galactoside (hyperin) | * |  |  | new |
| 20.45 | quercetin 3-glucuronide | * |  |  | new |
| 20.75 | quercetin 3-glucoside | * |  |  | new |
| 20.96 | lingnan 2 | * |  |  | new |
| 22.12 | methylpiceatannol glucoside | 419.1352 (M-H) | 419.1342 | 2.39 | old |
| 22.15 | kaempferol 3-galactoside | 449.1080(M+H) | 449.1088 | -1.78 | new |
| 22.61 | apigenin 7-glucoside | 433.1062 (M+H) | 433.1135 | -16.85 | new |
| 23.19 | kaempferol 3-glucoside (astragalin) | 471.0909 (M+H) | 471.0903 | 5.52 | both |
| 23.52 | isorhamnetin glucoside | 501.0983 (M+Na) | 501.1009 | -5.19 | both |
| 26.75 | methylpiceatannol (aglycon) | 257.0824 (M-H) | 257.0815 | 3.50 | old |
| 27.16 | kaempferol 3-rhamnoside | * |  |  | new |
| 29.91 | luteolin (aglycon) | * |  |  | new |
| 32.37 | monocoumaroylastragalin | 595.1445 (M+H) | 595.1448 | -0.50 | new |
| 33.09 | Unknown flavonoid | * |  |  | new |
| 33.68 | apigenin (aglycon) | 271.0659 (M+H) | 271.2448 | ? | new |
| 38.50 | 3,6-dicoumaroylastragalin der. | 763.1638 (M+23) | 763.1639 | -0.12 | both |
| 45.56 | 3,6-dicoumaroylastragalin | 763.1638 (M+23) | 763.1639 | -0.12 | both |

* Due to lack of ions detected in mass analyses, identification is based on retention time and LC–DAD spectrum
